# A High-Resolution Magic Angle Spinning NMR Study of the Enantiodiscrimination of 3,4-Methylenedioxymethamphetamine (MDMA) by an Immobilized Polysaccharide-Based Chiral Phase

**DOI:** 10.1371/journal.pone.0162892

**Published:** 2016-09-26

**Authors:** Juliana C. Barreiro, Márcio W. Paixão, Tiago C. Lourenço, Quezia B. Cass, Tiago Venâncio

**Affiliations:** 1 Department of Chemistry, Federal University of São Carlos, São Paulo, Brazil; 2 Scientific Director of Apex Science, Campinas, São Paulo, Brazil; National Cancer Institute at Frederick, UNITED STATES

## Abstract

This paper reports the investigation of the chiral interaction between 3,4-methylenedioxy-methamphetamine (MDMA) enantiomers and an immobilized polysaccharide-based chiral phase. For that, suspended-state high-resolution magic angle spinning nuclear magnetic resonance spectroscopy (^1^H HR-MAS NMR) was used. ^1^H HR-MAS longitudinal relaxation time and Saturation Transfer Difference (STD NMR) titration experiments were carried out yielding information at the molecular level of the transient diastereoisomeric complexes of MDMA enantiomers and the chiral stationary phase. The interaction of the enantiomers takes place through the aromatic moiety of MDMA and the aromatic group of the chiral selector by π-π stacking for both enantiomers; however, a stronger interaction was observed for the (*R*)-enantiomer, which is the second one to elute at the chromatographic conditions.

## Introduction

In chromatography, separation mechanisms are based not only on the solvated structure of the analyte and of the stationary phase but also on their tridimensional structure; therefore, enantiorecognition is a result of the sum of all these different interactions. Pinpointing these interactions at the molecular level is, however, an analytical challenge. In this context, saturation transfer difference nuclear magnetic resonance (STD NMR) spectroscopy has been successfully employed as a key technique to differentiate interactions at the supramolecular level [1]. For STD experiments, two ^1^H NMR spectra are acquired. The first spectrum corresponds to the ‘saturation on-resonance’ (STD_on_), in which the chiral stationary phase (CSP) ^1^H signals are selectively excited using a narrow NMR pulse that does not excite the enantiomer under analysis (the excitation is transferred throughout the CSP proton network by a mechanism called spin-diffusion, which is common for macromolecules). The second spectrum is a complementary control experiment and corresponds to the ‘saturation off-resonance’ (STD_off_), with a selective excitation applied to a point far from these CSP ^1^H signals. The difference spectrum (STD_diff_) is simply a subtraction of both spectra, and all STD data are reported showing two spectra; STD_diff_ and STD_off_ [1].

This approach can easily differentiate the interactions of each enantiomer in the transient diastereoisomeric complexes formed by the analyte and the chiral selector. For that, suspended-state high-resolution magic angle spinning nuclear magnetic resonance spectroscopy (^1^H HR-MAS NMR) can be used to mimic the chromatographic column conditions [2, 3].

Among the wide variety of chiral stationary phases (CSPs), polysaccharide-based coated phases exhibit a broad range of applicability to a variety of classes of compounds; however, there is a limited number of solvents that can be used without dissolving or swelling the chiral selector, and thus damaging the columns [4]. These limitations can compromise the analytical and preparative efficiencies mainly for larger scale preparative separations in which good solubility of the analyte is required to achieve high productivity. In the last three decades, the immobilization of polysaccharide derivatives on chromatographic supports has emerged as a major improvement in the technology and can help to solve this type of problem, allowing for a greater range of solvents to be used as eluent [4]. These chiral phases, however, need further mechanistic studies to adequately understand their chiral recognition ability under liquid chromatographic (LC) conditions.

**Fig 1:**
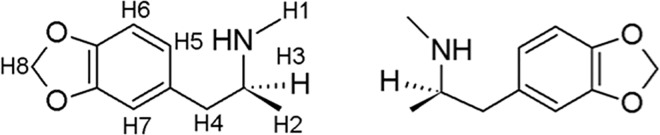
Structure of MDMA enantiomers.

To probe their chiral separation ability by ^1^H HR-MAS NMR, we selected the enantiomers of 3,4-methylenedioxy-methamphetamine (MDMA–Fig 1). Despite the large number of reported separations for chiral secondary amine derivatives, the enantioresolution of MDMA is a difficult task.

To date, derivatives of cyclodextrin have been the chiral selectors that yielded the best resolution of MDMA enantiomers by capillary electrophoresis and LC [5–11]. Moreover, MDMA is a chiral synthetic amphetamine used as a recreational racemic drug commonly known as “ecstasy” [12–14]. Toxicological studies have shown that the (*S*)-form is more active than the (*R*)-form in terms of “the degree and the disruptiveness of the induced intoxication” [9,12]. To proceed with toxicological studies, better analytical and multimilligram chiral separations are deemed necessary [11].

To meet this goal, the amylose tris(3-chlorophenylcarbamate) immobilized CSP yielded a high resolution at the analytical and semi-preparative LC scale, justifying the selection of MDMA as a probe for this CSP.

To throw light on the enantiorecognition mechanism of immobilized amylose based CSP towards the MDMA enantiomers, herein we fully discuss results obtained by ^1^H HR-MAS NMR. Furthermore, HR-MAS STD NMR experiments were used to determine the dissociation constant (*K*_D_) between the MDMA enantiomers and the used CSP.

## Materials and Methods

### Chemicals, reagents and samples

Acetonitrile of HPLC grade, was purchased from Merck (Darmstadt, Germany) and Mallinckrodt Baker (St. Louis, USA). Diethylamine (DEA) (99%) was purchased from Acros Organics (New Jersey, USA). MDMA was generously supplied by Prof. Dr. Regina Lúcia de Moraes Moreau (University of São Paulo-Faculty of Pharmaceutical Sciences). The analytical and semipreparative chiral columns were amylose 3-chlorophenylcarbamate-based (CHIRALPAK ID; 150 x 2.1 mm, 3 μm and 150 x 10 mm, 20 μm, respectively). Columns and the CHIRALPAK ID bulk stationary phase (CSP-ID) were furnished from Chiral Technologies Inc. (subsidiary of Daicel Corporation).

### Chromatographic conditions

The enantiomeric separation of MDMA was carried out by elution chromatography with stacked injection under mass overload condition using a mixture of acetonitrile and DEA (0.1%) as mobile phase at a flow rate of 5.0 mL.min^-1^. The preparative conditions were obtained after scaling up from the analytical conditions. MDMA was dissolved in the mobile phase solution at a concentration of 10 mg.mL^-1^. An injection volume of 0.5 mL of this solution was used, with a total of 27 injections in two cycles. The preparative HPLC system consisted of a Shimadzu LC-6AD pump, a Rheodyne 7725–018 injector fitted with a 500 μL loop, and a 10 AVvp variable wavelength UV-vis detector (λ = 270 nm) with a CBM SCL-10 Avp interface. Data acquisition was performed using the CLASS-VP software. The elution order was determined by optical rotation of the solutions (10 mg.mL^-1^) of the isolated enantiomers in either ethanol or water, (*S*)-(+)- and (*R*)-(-)-enantiomers. It is important to mention that the sign of the optical rotation of the enantiomers was inverted when determined in the chromatographic mobile phase (acetonitrile), compared to the ethanol and water solutions.

### NMR conditions

All NMR experiments were carried out at 400 MHz on a Bruker Avance III spectrometer equipped with a ^1^H HR-MAS probehead. The spectra were obtained using 1 mg of CSP-ID. All the stock and diluted solutions were prepared in ACN*d*_*3*_/DEA (100:0.1, *V*/*V*). The water signal at δ ≈ 2.0 ppm was suppressed. Samples were prepared in 4 mm ZrO_2_ rotors with a detection volume of 12 μL. The spectra were recorded at a spinning rate of 4kHz, at 298 K, under magic angle, properly adjusted by using anhydrous KBr. ^1^H STD NMR experiments were carried out using a total saturation time of 3 s. The STD spectrum of the analyte was acquired by subtraction of the spectrum with saturation (ON-resonance/300 Hz (δ = 0.75 ppm)) from one without saturation (OFF-resonance/12000 Hz (δ = 30.0 ppm)). STD titration experiments were performed increasing the analyte concentration from 5.0 to 78 mmol.L^-1^ and using different saturation times (0.5, 0.75, 1.0, 1.25, 1.5, 2.0, 2.5, 3.0, 4.0 and 5.0 s). The saturation pulse was applied by using a train of sixty 50 ms long Gaussian shaped pulses. Longitudinal relaxation times (*T*_1_) were also determined by inversion recovery in triplicate. The time interval between the 180° and 90° pulses was varied from 20 ms to 60 s with a relaxation delay of at least of 20 s between 13 experiments using with 16 scans each one. For the experiments, fresh solutions of each isolated MDMA enantiomer (1.0, 2.5, 5.0, 10 and 15 mg.mL^-1^) were prepared in a mixture of acetonitrile-*d*_*3*_ (ACN-*d*_*3*_) and DEA (100:0.1%, *V*/*V*) added to 1 mg of stationary phase (CHIRALPAK ID, 20 μm). The *T*_1_ experiment was repeated using 4 mg of CSP-ID with 15 mg.mL^-1^ of each enantiomer.

### Determination of K_D_

The dissociation constant (*K*_D_) was determined (*n* = 3) for free and bound (*R*) and (*S*)-enantiomers with 1mg of CSP-ID. For that, an isotherm saturation curve was experimentally designed through STD experiments fitting each enantiomer concentration (5.0, 13.0, 26.0, 52.0, and 78.0 mmol.L^-1^) at a saturation time of 3.0 s. The mean values (*n* = 3) were calculated by the following expression [15]:

$$A_{STD}=\frac{\alpha_{STD}\;[MDMA]}{K_{D}+[MDMA]}$$ (1)

Where α_STD_ is the maximum amplification factor. The *K*_D_ and α_STD_ values were obtained by linearization of the Eq 1, accordingly to a Lineweaver-Burk plot using QtiPlot software. For that, the *y*-axis corresponded to 1/A_STD_ and *x*-axis to 1/[MDMA].

## Results and Discussion

**Fig 2:**
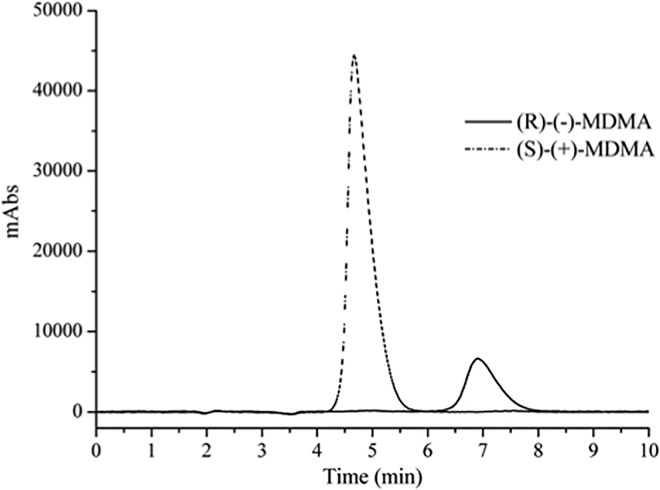
Elution order of MDMA enantiomers in ACN*d*_*3*_/DEA (100:0.1, v/v). Flow rate of 5.0 mL.min^-1^, λ = 270 nm and injection volume of 0.5 mL.min^-1^.

For ^1^H HR-MAS NMR experiments, first it is necessary to obtain the enantiomers in a high degree of enantiomeric purity. The MDMA enantiomers (135 mg) were separated by a total of 27 continuous injections (2.64 h), providing a throughput of almost 577 mg.d^-1^ for the enantiomers. The first (*S*)-enantiomer (63.1 mg) and the second (*R*)-enantiomer (64.0 mg) were both obtained in recoveries higher than 93%. Enantiomeric purity higher than 99.9% was obtained for both enantiomers (Fig 2).

**Fig 3:**
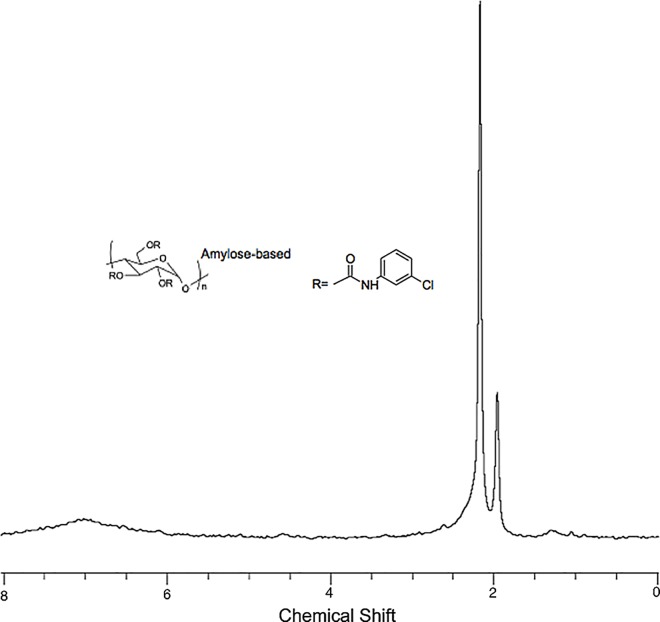
^1^H HR-MAS NMR spectrum of CHIRALPAK ID (Amylose 3-chlorophenylcarbamate-selector). 1 mg suspended in 12 μL of ACN*d*_*3*_/DEA (100:0.1, v/v).

The ^1^H HR-MAS NMR spectrum of the CSP-ID at the chromatographic mobile phase used is illustrated in Fig 3. The signal broadening at 6 and 8 ppm is due to the limited molecular mobility causing a short *T*_2_^*^.

As in the coated polysaccharide phases, hydrogen bonding, π-π, dipole-dipole stacking, and steric inclusion are the most likely interactions between the chiral selector and the enantiomers during the separation process.

**Fig 4:**
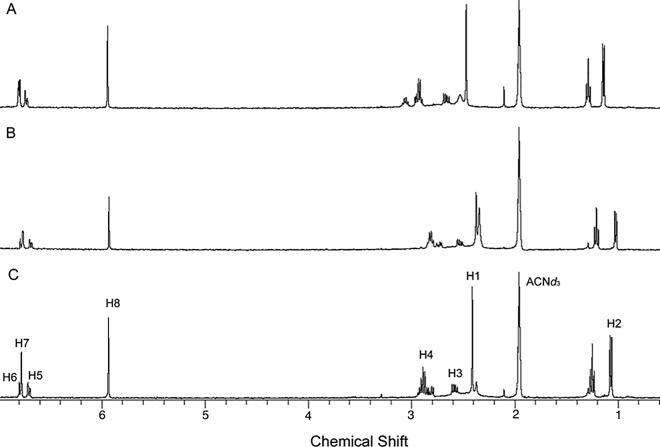
^1^H HR-MAS NMR of (A) (*R*)-enantiomer (13.0 mmol.L^-1^), (B) (*S*)-enantiomer (13.0 mmol.L^-1^), and (C) racemic mixture (13.0 mmol.L^-1^) in the presence of 1 mg of CSP suspended in ACN*d*_*3*_/DEA (100:0.1, v/v).

The NMR spectra obtained for the (*R*)-enantiomer (A) and (*S*)-enantiomer (B) and the racemic mixture (C) in the presence of CSP-ID are illustrated in Fig 4.

**Fig 5:**
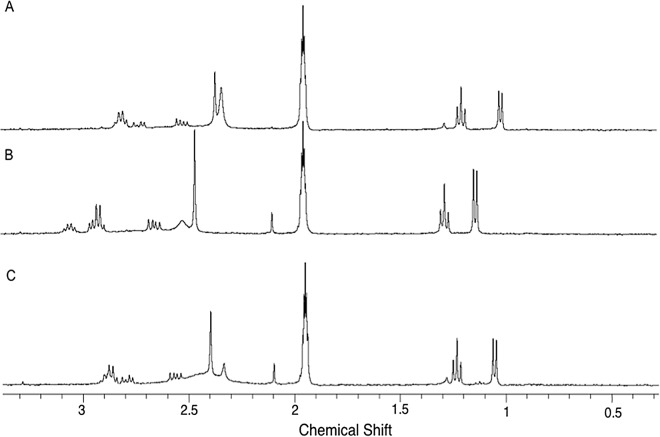
Expanded Spectra ^1^H HR-MAS NMR of (A) (*S*)-enantiomer (13.0 mmol.L^-1^), (B) (*R*)-enantiomer (13.0 mmol.L^-1^), and (C) racemic mixture (13.0 mmol.L^-1^) in the presence of 1 mg of CSP-ID suspended in ACN*d*_*3*_/DEA (100:0.1, v/v).

The spectrum of the racemic mixture (Fig 4C) shows the chemical shift for the protons located at H1 (δ = 2.55 ppm), H2 (δ = 1.14–1.13 ppm), H3 (δ = 2.65–2.80 ppm), H4 (δ = 2.91–3.31 ppm), H5 (δ = 6.60–6.71 ppm), H6 (δ = 6.79–6.71 ppm), H7 (δ = 6.75 ppm) and H8 (δ = 5.96 ppm), as assigned at the MDMA chemical structure (Fig 1). Changes in chemical shift can be perceived for the protons located at position H2, H3 and H4 when the spectra of Fig 4 are compared. These changes are better observed in the expanded spectra of both enantiomers shown in Fig 5. Major differences in chemical shift are observed for the (*R*)-enantiomers when compared to the other two spectra (B and C). This is an indicative that the (*R*)-enantiomer strongly interacts with CSP.

The *T*_1_ ratio values [16] of the (*S*)- and (*R*)-enantiomers in homogeneous solution and in CSP-ID suspension are summarized on Table 1. The *T*_1_ values show that the spin-lattice relaxation times are shorter for the enantiomers in the diastereoisomeric complex, especially for the protons of the (*R*)-enantiomer, compared to analogous relaxation times for the dissolved species, indicating a binding affinity towards the CSP-ID. These results indicate that there is a difference in the dynamic behavior of the enantiomers when bound to CSP-ID, suggesting that the configuration of (*R*)-MDMA is the one that allows the closest interaction to the chiral selector, decreasing its correlation time. The ratio of *T*_1_ values obtained at 4 mg of CSP-ID in comparison with the values obtained at 1 mg (Table 1) is significantly lower for all the protons, and somehow the values are closer to the (*S/R*) ratio in solution. This effect can be probably caused by a shift in the equilibrium of the diastereoisomeric complex favoring the less retained (*S*)-enantiomer, and losing enantioselectivity.

**Table 1:** *T*_1_(s) values (mean *n* = 3) for the (*S*)- and (*R*)-MDMA (78.0 mmol.L^-1^) enantiomers in solution of ACN*d*_*3*_/DEA (100:0.1, v/v) and with CSP-ID.

^1^H	(*S*)/(*R*) (solution)	(*S*)-MDMA (w/CSP-ID)^a^	(*R*)-MDMA (w/CSP-ID)^a^	(*S*)/(*R*) (w/CSP-ID)^a^	(*S*)-MDMA (w/CSP-ID)^b^	(*R*)-MDMA (w/CSP-ID)^b^	(*S*)/(*R*) (w/CSP-ID)^b^
H6, H5, H7	1.22	3.17	1.84	1.72	3.23	2.57	1.25
H8	1.29	3.21	1.97	1.67	2.85	2.68	1.06
H3	-	-	1.81	-	-	1.58	-
H4	1.19	2.39	1.46	1.64	1.42	1.49	0.95
H1	1.10	2.62	1.79	1.46	1.79	1.77	1.01
H2	1.49	1.62	1.00	1.62	1.31	1.49	0.88

^a^1 mg of CSP-ID suspended in 12 μL of each enantiomers (78.0 mmol.L^-1^) in solution of ACN*d*_*3*_/DEA (100:0.1, v/v).

^b^4 mg of CSP-ID suspended in 12 μL of each enantiomers (78.0 mmol.L^-1^) in solution of ACN*d*_*3*_/DEA (100:0.1, v/v).

*T*_1_ values have been already used to designate the most retained enantiomer [3, 2, 16].

### STD Experiments

The ^1^H STD HR-MAS NMR spectra essentially reveal the analyte’s proton signals closer to the sorbent, since they are more sensitive to magnetization transfer. As a result, the proton with the strongest interaction with the stationary phase gives the most intense signals [17]. It was not possible to observe the STD signals at short saturation times (0.5 to 2.5 s). Thus, the value of 3.0 s was selected for the STD experiments.

**Fig 6:**
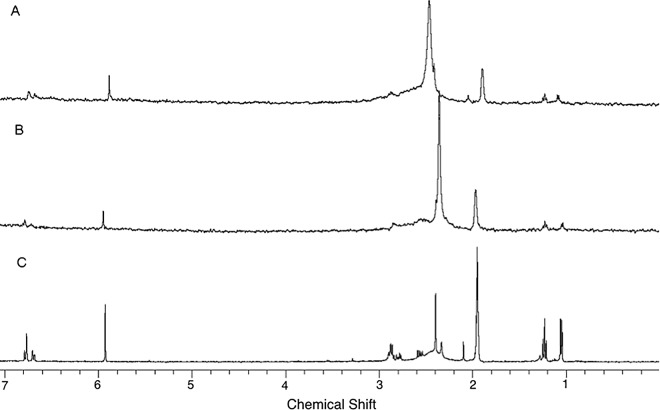
^1^H STD HR-MAS NMR for: (A) (*R*)-MDMA (13.0 mmol.L^-1^), on resonance, (B) (*S*)-MDMA (13.0 mmol.L^-1^) ON-resonance and (C) MDMA racemic mixture (13.0 mmol.L^-1^) OFF-resonance.

The comparison of the spectra of Fig 6A and 6B with the spectra at of Fig 6C reveals that the later has a new set of proton signals corresponding to H2 (-CH_3_), H5-H6 (aromatic), and H8 (-CH_2_). In the spectrum of (*S*)-MDMA-CSP-ID (Fig 6B), the aromatic proton signals are of lower intensity when compared to those of (*R*)- MDMA-CSP-ID (Fig 6A).

**Fig 7:**
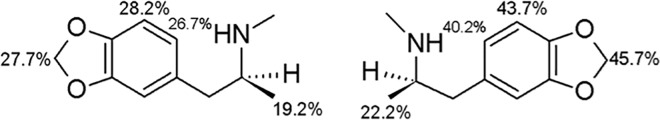
The structures of MDMA enantiomers with the percentage of signals intensity as obtained from the STD experiments. The first structure corresponds to the (*S*)-MDMA and the second to (*R*)-MDMA.

The STD signals were normalized with respect to the registered signal of highest intensity (ACN*d*_3_ –solvent signal), which was assigned as 100%. The percentage signal intensities are given in Fig 7.

The protons at the positions H8 (45.7%) and H6 (43.7%) of the (*R*)- MDMA enantiomer showed the highest interactions, corroborating the *T*_1_ values as well as, the obtained chromatographic retention factors. Thus, it can be assumed that the aromatic ring is the most important moiety for the molecular enantiorecognition process.

Here it is worth noting that the chiral selector is based on 3-chlorophenyl derivatives that furnish a more effective π-π interactions.

### Determination of K_D_

**Fig 8:**
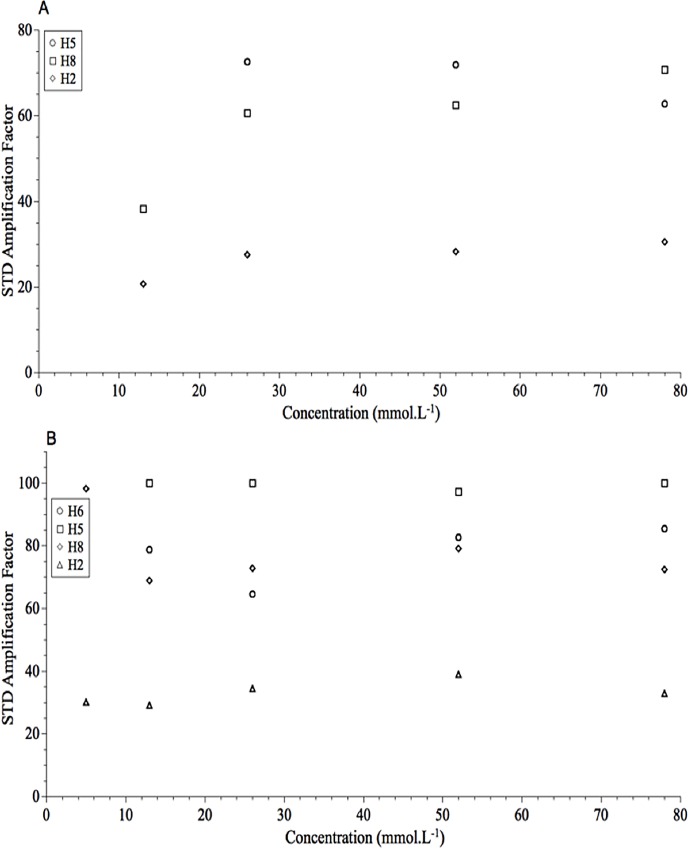
Graphic of STD amplification factor (mean *n* = 3) versus concentration of enantiomer at 3.0 s of saturation time. (A) (*S*)-MDMA and (B) (*R*)-MDMA.

Chromatographic chiral recognition is a result of energy differences between the formations of the two transient diastereomeric complexes. Conceptually, it is similar to enantiomeric differentiation in biological process [18]. STD experiments have been commonly employed to determine *K*_D_ values of the protein-ligand complexes by measuring the equilibrium concentration of the free and bound species [19]. The isotherms fitted from the STD experiments (Fig 8) for both enantiomers were used to calculate K_D_; from these experiments, it was possible to estimate the equilibrium constants (K_on_/K_off_) involved in the formation of the transient diastereoisomeric complex of (*R*)-MDMA and CSP-ID. It is important to note that the STD NMR titrations are strongly dependent of experimental conditions such as the, saturation time, relative concentration of the species, and monitored protons [19].

The results herein described extend the applicability of STD NMR spectroscopy for unrevealing the equilibrium constant for the weak interactions involved in enantioselective chromatography. As far as we could ascertain, this is the first time such experiments have been used to determine *K*_D_ for a transient diastereoisomer, which is of particular relevance in designing new chiral selectors for liquid chromatography.

The difficulty to estimate *K*_D_ for the first enantiomers (Fig 8A) is due to the variability of the STD data at low concentrations. This behavior can be related to the poor integration of the proton signals in the spectra, even though at 256 scans. In addition, the MAS conditions and the heterogeneity of the sample contribute to the poor estimation of *K*_D_ especially for the (*S*)-enantiomer, which is the one with the weakest interaction. The mean (*n* = 3) *K*_D_ value for the (*R*)-enantiomer was of 1.52 ± 0.94 mmol.L^-1^, calculated from the plot illustrated in Fig 8B.

The low *K*_D_ value obtained for the (*R*)-enantiomer implies a weak interaction [1] in the formation of the diastereoisomer, even though, it reflects the obtained elution order and corroborates the difficulty in measuring the *K*_D_ value for the (*S*)-enantiomer.

## Conclusions

The CHIRALPAK-ID stationary phase was fully able to separate MDMA enantiomers by stack injections under mass overload conditions with high throughput, yielding the enantiomers with high enantiomeric purity for the HR-MAS-NMR experiments. Amongst the MAS procedures, the STD NMR experiment was the one that provided more information about the molecular interactions of the MDMA enantiomers with the CSP-ID. As disclosed by these experiments, the interaction of the enantiomers takes place through the aromatic moiety of the MDMA and the aromatic group of the chiral selector (3-chlorophenyl group) by π-π stacking, among others. It is important to note that, although both enantiomers can be discriminated through this moiety, a strong interaction is observed for the (*R*)-enantiomer, thus justifying the elution order.
